# Comparison of the Diagnostic Performance of ACR TI-RADS and EU-TI-RADS for the Detection of Thyroid Malignancy: A Retrospective Study

**DOI:** 10.7759/cureus.113675

**Published:** 2026-07-30

**Authors:** Abdelwahed S Abougazia, Ban Abdullah, Ahmed Abdelmoneim, Amal Mahran, Tamer Ali, Maryam M Alemadi, Soliena Alnakawa, Fisal Haritani, Rana Bakkar, Hussameddin Alali, Abdulla Alnaama, Maysaa Ashkanani, Ahmed Sameer Alnuaimi

**Affiliations:** 1 Radiology, Primary Health Care Corporation (PHCC), Doha, QAT; 2 Clinical Imaging, Hamad Medical Corporation, Doha, QAT; 3 Family Medicine, Primary Health Care Corporation (PHCC), Doha, QAT; 4 Clinical Research, Primary Health Care Corporation (PHCC), Doha, QAT

**Keywords:** acr ti-rads, diagnostic accuracy, eu-ti-rads, fine-needle aspiration cytology, thyroid cancer detection, thyroid malignancy, thyroid nodules, thyroid ultrasound, ultrasound risk stratification

## Abstract

Objective

The objective of this study was to compare the diagnostic performance of the American College of Radiology Thyroid Imaging Reporting and Data System (ACR TI-RADS) and the European Thyroid Imaging Reporting and Data System (EU-TI-RADS) in differentiating benign from malignant thyroid nodules using guideline-based fine-needle aspiration recommendation as the test-positive criterion, and to evaluate demographic and sonographic predictors of thyroid malignancy in a real-world clinical cohort.

Materials and methods

This retrospective diagnostic accuracy study included thyroid nodules assessed by ultrasound followed by ultrasound-guided fine-needle aspiration cytology (FNAC), with histopathology used as the reference standard for surgically excised nodules. Ultrasound images were independently reviewed by three experienced consultant radiologists blinded to cytological and histopathological outcomes, with consensus used for disagreements. The definitive cohort comprised 809 nodules: 652 benign and 157 malignant. Nodules without a definitive outcome, including nonoperated Bethesda III/IV cases, were excluded from the primary analysis. Bethesda I nodules remained excluded because they were nondiagnostic. Diagnostic performance was calculated using 2 × 2 contingency tables, including sensitivity, specificity, positive predictive value (PPV), negative predictive value (NPV), accuracy, likelihood ratios, and diagnostic OR (DOR). Paired comparisons were performed using McNemar testing. Multivariable logistic regression was performed to identify independent predictors of malignancy. Sensitivity analyses were performed by classifying excluded nonoperated Bethesda III/IV nodules as benign and malignant in separate scenarios.

Results

The definitive cohort comprised 809 nodules, including 652 benign nodules and 157 malignant nodules. ACR TI-RADS correctly identified 135 of 157 malignant nodules (sensitivity 85.99%) and correctly excluded 481 of 652 benign nodules (specificity 73.77%). EU-TI-RADS correctly identified 142 of 157 malignant nodules (sensitivity 90.45%) but correctly excluded only 233 of 652 benign nodules (specificity 35.74%). ACR TI-RADS demonstrated higher PPV (44.12% vs 25.31%), NPV (95.63% vs 93.95%), overall accuracy (76.14% vs 46.35%), and DOR (17.26 vs 5.26). EU-TI-RADS had slightly higher sensitivity than ACR TI-RADS (McNemar p = 0.046), whereas ACR TI-RADS had significantly higher specificity (McNemar p < 0.001). Sensitivity analyses including nonoperated Bethesda III/IV nodules yielded similar results. Multivariable logistic regression identified hypoechogenicity, solid or almost entirely solid composition, and taller-than-wide orientation as independent predictors of malignancy, while irregular or lobulated margins and microcalcifications showed quasi-complete separation because of their strong association with malignant nodules.

Conclusions

Both systems demonstrated high sensitivity and high NPV. In this primary healthcare cohort, ACR TI-RADS provided substantially higher specificity and overall accuracy, suggesting greater potential to reduce unnecessary FNAC, whereas EU-TI-RADS offered marginally higher sensitivity. Hypoechogenicity, solid or almost entirely solid composition, and taller-than-wide orientation were independent predictors of malignancy. Irregular or lobulated margins and microcalcifications were also strongly associated with malignancy but showed quasi-complete separation.

## Introduction

Thyroid nodules are frequently detected in clinical practice, with higher rates in women and increasing recognition due to the widespread use of ultrasound [1]. Although most thyroid nodules are benign, a clinically significant minority are malignant and need reliable imaging-based risk stratification. Ultrasound features commonly associated with malignancy include predominantly solid composition, hypoechogenicity, irregular or lobulated margins, taller-than-wide orientation, and punctate echogenic foci (microcalcifications), although individual features have variable predictive values when considered in isolation [2,3].

Multiple ultrasound-based classification systems are used for consistent risk stratification and reduction of unnecessary fine-needle aspiration cytology (FNAC). The American College of Radiology Thyroid Imaging Reporting and Data System (ACR TI-RADS) and the European Thyroid Imaging Reporting and Data System (EU-TI-RADS) are among the most widely used systems, and they link structured ultrasound categories to size-based FNAC thresholds but differ in the weighting of ultrasound features and category definitions [4,5].

Prior studies comparing ACR TI-RADS and EU-TI-RADS showed a consistent trade-off between sensitivity and specificity, with EU-TI-RADS generally achieving higher sensitivity at the expense of increased FNAC utilization, while ACR TI-RADS showed higher specificity and biopsy efficiency [6-8]. However, relatively few studies have directly compared these systems using guideline-based FNAC recommendation as the diagnostic threshold and a pragmatic composite reference standard. Furthermore, most available evidence originates from tertiary referral or specialist endocrine centers, where the case mix may be influenced by referral bias and a higher prevalence of suspicious or malignant nodules.

Evidence from primary healthcare settings remains limited. The present study adds real-world data from a large Primary Health Care Corporation (PHCC)-based primary healthcare cohort and evaluates both systems using blinded retrospective image review and a pragmatic composite outcome standard. Importantly, thyroid ultrasound evaluation and TI-RADS classification were performed in the primary healthcare setting, with patients subsequently referred for FNAC to a secondary hospital, reflecting a real-world primary-to-secondary care diagnostic pathway. Such a model may better represent the spectrum of thyroid nodules encountered in routine clinical practice and allow evaluation of guideline-based biopsy decisions outside specialized tertiary centers.

We hypothesized that EU-TI-RADS would demonstrate higher sensitivity for thyroid malignancy detection than ACR TI-RADS, whereas ACR TI-RADS would provide greater specificity and reduce unnecessary biopsy recommendations. The aim of this study was to compare the diagnostic performance and clinical implications of both classification systems in a large cohort of thyroid nodules with definitive cytological or histopathological outcomes. This study provides additional evidence regarding diagnostic accuracy, biopsy recommendations, and missed malignancy patterns between the two systems.

Accordingly, the objectives of this study were (1) to compare the diagnostic performance of ACR TI-RADS and EU-TI-RADS in differentiating benign from malignant thyroid nodules using guideline-based FNAC recommendation as the test-positive criterion and (2) to evaluate demographic and sonographic predictors of thyroid malignancy in a real-world clinical cohort.

## Materials and methods

Study design, setting, and population

This retrospective diagnostic accuracy study was conducted using electronic medical records from two large healthcare institutions. All patients who underwent thyroid ultrasound followed by ultrasound‑guided FNAC of a focal thyroid nodule during the study period from November 2023 to November 2024 were eligible for inclusion. Thyroid ultrasound evaluations were performed at primary care centers, and nodules requiring biopsy were referred to a secondary hospital for FNAC, reflecting the primary-to-secondary care referral pathway in our healthcare system.

Ethics approval

This study was reviewed and approved by the PHCC Institutional Review Board (IRB), Doha, Qatar (IRB reference no. BUHOOTH-D-24-00087). As a retrospective analysis of existing clinical, imaging, cytological, and histopathological data with no direct patient contact, the IRB waived the requirement for informed consent. Prospective trial registration was not applicable.

Ultrasound assessment and TI-RADS classification

Thyroid ultrasound examinations were performed using high-resolution ultrasound systems equipped with linear-array transducers (10-15 MHz) in keeping with routine departmental practice. Standard thyroid imaging protocols were utilized, including transverse and longitudinal assessment of both lobes and the isthmus. For each nodule, standard sonographic features were recorded, including location, maximal diameter, composition, echogenicity, shape and orientation, margin characteristics, calcification pattern, and additional features such as vascularity, halo, and suspected extrathyroidal extension where available.

All available thyroid ultrasound images were retrospectively reviewed independently by three consultant radiologists with 15-20 years of experience in thyroid imaging. The reviewers assessed the documented nodule images and sonographic criteria and classified each nodule according to both ACR TI-RADS and EU-TI-RADS. The reviewers were blinded to cytological and histopathological outcomes at the time of image assessment. In cases of disagreement, a final category was assigned by consensus after joint review. Consensus scoring was defined as agreement between participating radiologists regarding the final TI-RADS category assignment after independent review of the available ultrasound images and reports. Because the final study dataset retained only consensus-assigned TI-RADS categories and individual reader-level classifications were not preserved, formal interobserver agreement analysis (e.g., kappa statistics) could not be performed.

To minimize incorporation and interpretation bias, the retrospective TI-RADS classification was performed independently of the original clinical report and independently of the pathological outcome. Guideline-specific recommendations for FNAC were recorded for all nodules. When available, cervical lymph node regions were also assessed during ultrasound examinations, and suspicious lymphadenopathy was documented according to institutional reporting standards.

Nodule selection and reference standard

When more than one thyroid nodule was present in the same patient, any nodule that underwent FNAC and had complete ultrasound features and definitive cytological or histopathological outcome data was eligible for inclusion. If multiple eligible nodules were present in the same patient, each nodule was analyzed as a separate observation; therefore, the unit of analysis was the nodule rather than the patient. Cases with incomplete imaging data or unavailable definitive cytological/histopathological outcomes were excluded from the final diagnostic accuracy analysis.

FNAC results were reported using the Bethesda System for Reporting Thyroid Cytopathology [9]. A pragmatic composite reference standard was used. For surgically excised nodules, final histopathology was considered the definitive outcome. For nonoperated nodules, diagnostic FNAC was used for outcome classification: Bethesda II was considered benign, whereas Bethesda V and VI were considered malignant. Nonoperated nodules with indeterminate cytology, including Bethesda III and IV, were excluded from the primary diagnostic accuracy analysis because definitive benign or malignant classification could not be established without surgical histopathology. Bethesda I nodules were excluded because they were nondiagnostic. Operated nodules were classified according to final histopathology irrespective of their cytology category.

Statistical analysis

Categorical variables were reported as counts and percentages, and group differences were assessed using the chi-square or Fisher’s exact tests, as appropriate. To evaluate independent predictors of thyroid malignancy, multivariable logistic regression analysis was performed using the final diagnostic outcome, benign vs malignant, as the dependent variable. Predictor variables were selected based on clinical relevance and univariate sonographic associations and included age, sex, solid or almost entirely solid composition, hypoechogenicity, taller-than-wide orientation, irregular or lobulated margins, and microcalcifications. ORs with 95% CIs were calculated. Variables showing quasi-complete separation were identified, and stable conventional OR estimates were not reported for these variables.

Diagnostic performance was assessed using 2 × 2 contingency tables, with guideline-based FNAC recommendation considered the test-positive criterion. Sensitivity, specificity, positive predictive value (PPV), negative predictive value (NPV), overall accuracy, positive likelihood ratio (LR+), negative likelihood ratio (LR-), and diagnostic OR (DOR) were calculated for ACR TI-RADS and EU-TI-RADS. Ninety-five percent CIs were calculated for proportions using the Wilson method. Because both classification systems were applied to the same nodules, paired comparisons were performed using McNemar testing. McNemar testing was performed separately in malignant nodules to compare sensitivity and in benign nodules to compare specificity. This approach was used because sensitivity is determined only among malignant nodules, whereas specificity is determined only among benign nodules; therefore, discordant paired recommendations were assessed within the relevant outcome subgroup for each metric. Ordinal discriminatory performance was additionally explored using the numerical TI-RADS categories to calculate the area under the receiver operating characteristic curve (AUC). Sensitivity analyses were performed by reclassifying excluded nonoperated Bethesda III/IV nodules as benign in one scenario and malignant in another scenario to evaluate the robustness of the primary findings. Statistical significance was set at a two-sided p-value of <0.05.

Information regarding previous neck irradiation, familial thyroid cancer syndromes, or other hereditary cancer predisposition conditions was not systematically available and therefore was not included in the analysis.

Reporting standards

**Figure 1:**
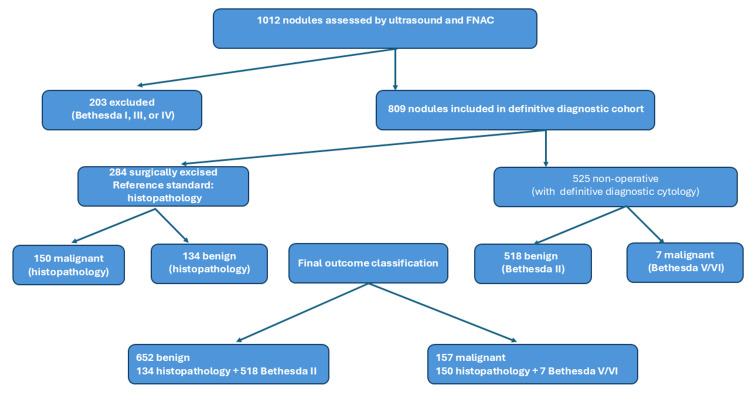
Flow diagram of thyroid nodule selection and reference standard classification. Of 1012 nodules assessed by ultrasound and FNAC, 203 nodules with nondiagnostic or indeterminate cytology (Bethesda I, III, or IV) were excluded. The definitive diagnostic cohort comprised 809 nodules, including 284 surgically excised nodules classified by histopathology and 525 nonoperative nodules classified by definitive cytology. Overall, the final cohort included 652 benign and 157 malignant nodules. FNAC, fine-needle aspiration cytology

The Standards for Reporting Diagnostic Accuracy Studies (STARD) framework was used to guide reporting of participant selection, index test interpretation, reference standard definition, flow of included and excluded nodules, and diagnostic accuracy estimates [10]. The study flow is illustrated in Figure 1.

## Results

Study cohort and cytology/histopathology spectrum

A total of 203 nodules were excluded from the definitive diagnostic accuracy cohort because of nondiagnostic or indeterminate cytology, including 129 Bethesda I, 73 Bethesda III, and one Bethesda IV nodule. After application of the composite reference standard, 809 nodules were included in the definitive diagnostic accuracy cohort, comprising 652 benign nodules and 157 malignant nodules (Table 1). Patient and nodule selection, including surgical and nonoperative reference standard pathways and exclusions, is summarized in Figure 1. The mean age was 46.36 ± 11.18 years, with a median of 45 years and a range of 19-82 years. The definitive diagnostic cohort comprised 809 nodules. Demographic variables were summarized at the nodule level because the unit of analysis was the nodule rather than the individual patient; therefore, patients with more than one eligible biopsied nodule could contribute more than one observation. FNAC results were categorized according to the Bethesda System for Reporting Thyroid Cytopathology. Only nodules with definitive benign or malignant outcomes were included in the primary diagnostic accuracy analysis. Seventy-four nonoperated nodules with indeterminate cytology (73 Bethesda III and 1 Bethesda IV) were excluded from the primary analysis owing to the absence of definitive histopathological confirmation. Bethesda I nodules (129 nodules) remained excluded as nondiagnostic.

**Table 1:** Definitive diagnostic cohort characteristics (n = 809). Values are presented as numbers unless otherwise stated. Demographic variables are summarized at the nodule level because the unit of analysis was the nodule rather than the individual patient.

Characteristic	Value
Definitive diagnostic accuracy cohort	809 nodules
Benign outcome	652 (80.6%)
Malignant outcome	157 (19.4%)
Age, mean ± SD	46.36 ± 11.18 years
Age, median (range)	45 years (19-82)
Female nodules	622 (76.9%)
Male nodules	187 (23.1%)
Excluded nonoperated indeterminate nodules	74 (73 Bethesda III and 1 Bethesda IV)

TI-RADS distribution and FNAC recommendations

The distribution of EU-TI-RADS scores and FNAC recommendations, and ACR TI-RADS categories and FNAC recommendations, is shown in Table 2 and Table 3. According to EU-TI-RADS, most nodules were classified as EU-TI-RADS 3 (638/809, 78.9%), followed by EU-TI-RADS 5 (120/809, 14.8%) and EU-TI-RADS 4 (41/809, 5.1%).

**Table 2:** Distribution of EU-TI-RADS and ACR TI-RADS categories in the definitive diagnostic cohort (n = 809). Percentages are calculated using the definitive diagnostic cohort. ACR TI-RADS, American College of Radiology Thyroid Imaging Reporting and Data System; EU-TI-RADS, European Thyroid Imaging Reporting and Data System

Guideline	Category	n (%)
EU-TI-RADS	EU-TI-RADS 2	10 (1.2)
EU-TI-RADS	EU-TI-RADS 3	638 (78.9)
EU-TI-RADS	EU-TI-RADS 4	41 (5.1)
EU-TI-RADS	EU-TI-RADS 5	120 (14.8)
ACR TI-RADS	TR1: 0 points (benign)	10 (1.2)
ACR TI-RADS	TR2: 2 points (not suspicious)	317 (39.2)
ACR TI-RADS	TR3: 3 points (mildly suspicious)	289 (35.7)
ACR TI-RADS	TR4: 4-6 points (moderately suspicious)	85 (10.5)
ACR TI-RADS	TR5: ≥7 points (highly suspicious)	108 (13.3)

**Table 3:** FNAC recommendations according to EU-TI-RADS and ACR TI-RADS thresholds (n = 809). The FNAC recommendation was defined according to the size-based and risk-based thresholds specified in each guideline. ACR TI-RADS, American College of Radiology Thyroid Imaging Reporting and Data System; EU-TI-RADS, European Thyroid Imaging Reporting and Data System; FNAC, fine-needle aspiration cytology

Guideline	FNAC recommendation	n (%)
EU-TI-RADS	Yes	561 (69.3)
EU-TI-RADS	No	248 (30.7)
ACR TI-RADS	Yes	306 (37.8)
ACR TI-RADS	No	503 (62.2)

According to ACR TI-RADS, most nodules were categorized as TR2 or TR3, representing 317 (39.2%) and 289 (35.7%) nodules, respectively, while fewer nodules were classified as TR4 or TR5. FNAC recommendation was more frequent with EU-TI-RADS (561/809, 69.3%) than with ACR TI-RADS (306/809, 37.8%).

Sonographic features: benign vs malignant

**Figure 2:**
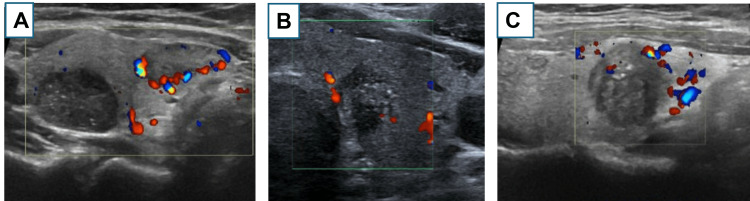
Representative ultrasound images (A-C) of classic high-suspicion malignant thyroid nodules. Panels A and B show transverse views, while panel C shows a sagittal view. The nodules demonstrate solid composition, marked hypoechogenicity, irregular margins, taller-than-wide orientation, and punctate echogenic foci. These appearances correspond to TR5/EU-TI-RADS 5 categories and illustrate typical imaging patterns that appropriately meet guideline-based criteria for FNAC. EU-TI-RADS, European Thyroid Imaging Reporting and Data System; FNAC, fine-needle aspiration cytology

The distribution of key sonographic features is summarized in Table 4. Comparison of ultrasound features between benign and malignant nodules demonstrated that malignancy was significantly associated with predominantly solid composition (solid or almost entirely solid), hypoechogenicity or marked hypoechogenicity, taller-than-wide orientation, lobulated or irregular margins, and microcalcifications. In contrast, benign nodules were predominantly isoechoic, exhibited smooth margins, and lacked microcalcifications. Representative examples of classic high-suspicion malignant thyroid nodules demonstrating solid composition, marked hypoechogenicity, irregular margins, taller-than-wide orientation, and punctate echogenic foci, corresponding to TR5/EU-TI-RADS 5 categories, are shown in Figure 2A-2C.

**Table 4:** Sonographic features of benign and malignant nodules in the definitive diagnostic cohort (n = 809). Data are presented as numbers (percentages) within each diagnostic group. Percentages are calculated within columns using the total number of benign nodules (n = 652) and malignant nodules (n = 157) as denominators. p-values represent comparisons across all categories within each sonographic feature using Pearson chi-square tests or Fisher’s exact tests, as appropriate. Test statistics are reported for each feature and correspond to the overall comparison between benign and malignant groups. Fisher’s exact ORs are presented for binary variables with small cell counts. Infinite ORs indicate that the feature was observed exclusively in malignant nodules. The definitive diagnostic cohort comprised 809 nodules.

Feature	Category	Benign, n (%)	Malignant, n (%)	Test statistic	p-value
Composition	Almost entirely cystic	7 (1.1)	0 (0.0)	χ² (4) = 107.94	<0.001
	Almost entirely solid	153 (23.5)	36 (22.9)		
	Cystic	3 (0.5)	0 (0.0)		
	Mixed solid-cystic	341 (52.3)	23 (14.6)		
	Solid	148 (22.7)	98 (62.4)		
Echogenicity	Anechoic	10 (1.5)	0 (0.0)	χ² (4) = 429.88	<0.001
	Hyperechoic	16 (2.5)	0 (0.0)		
	Hypoechoic	25 (3.8)	101 (64.3)		
	Isoechoic	601 (92.2)	42 (26.8)		
	Markedly hypoechoic	0 (0.0)	14 (8.9)		
Shape	Irregular	0 (0.0)	12 (7.6)	χ² (2) = 52.51	<0.001
	Oval	647 (99.2)	142 (90.4)		
	Round	5 (0.8)	3 (1.9)		
Orientation	Taller than wide	1 (0.2)	17 (10.8)	Fisher’s exact OR = 79.05	<0.001
	Wider than tall	651 (99.8)	140 (89.2)		
Margin	Ill-defined	219 (33.6)	38 (24.2)	χ² (3) = 265.03	<0.001
	Irregular	0 (0.0)	28 (17.8)		
	Lobulated	0 (0.0)	31 (19.7)		
	Smooth	433 (66.4)	60 (38.2)		
Microcalcification	Absent	652 (100.0)	57 (36.3)	χ² (1) = 473.86	<0.001
	Present	0 (0.0)	100 (63.7)		
Macrocalcification	Absent	595 (91.3)	123 (78.3)	χ² (1) = 21.14	<0.001
	Present	57 (8.7)	34 (21.7)		
Rim calcification	Absent	647 (99.2)	150 (95.5)	Fisher’s exact OR = 6.04	0.003
	Present	5 (0.8)	7 (4.5)		
Extrathyroidal extension	Present (capsular bulge)	0 (0.0)	4 (2.5)	Fisher’s exact OR = ∞	0.001
	No extrathyroidal extension	652 (100.0)	153 (97.5)		

Independent predictors of thyroid malignancy

Multivariable logistic regression analysis was performed to identify independent predictors of thyroid malignancy after adjustment for demographic and sonographic variables. Younger age was independently associated with malignancy (OR 0.96 per year increase, 95% CI 0.93-1.00, p = 0.028), whereas male sex did not remain significant after adjustment (OR 1.95, 95% CI 0.88-4.32, p = 0.099). Hypoechogenicity was a strong independent predictor of malignancy (OR 13.74, 95% CI 6.03-31.27, p < 0.001). Solid or almost entirely solid composition remained independently associated with malignancy (OR 2.55, 95% CI 1.07-6.10, p = 0.035). Taller-than-wide orientation demonstrated the strongest estimable association with malignancy (OR 34.43, 95% CI 2.55-465.85, p = 0.008). Irregular or lobulated margins and microcalcifications exhibited quasi-complete separation because these features were absent among benign nodules and highly concentrated among malignant nodules; therefore, stable conventional OR estimates could not be reliably obtained. The results of the multivariable model are summarized in Table 5.

**Table 5:** Multivariable logistic regression analysis of independent predictors of thyroid malignancy in the definitive diagnostic cohort (n = 809). Regression model parameters are reported as ORs with corresponding 95% CIs and p-values derived from the multivariable logistic regression model. The dependent variable was the final diagnostic outcome, categorized as benign or malignant. Predictor variables included age, sex, hypoechogenicity, solid or almost entirely solid composition, taller-than-wide orientation, irregular or lobulated margins, and microcalcifications. Solid or almost entirely solid composition included nodules classified as solid or almost entirely solid. Hypoechogenicity included hypoechoic and markedly hypoechoic nodules. Irregular or lobulated margins and microcalcifications demonstrated quasi-complete separation because these features were absent among benign nodules and strongly concentrated among malignant nodules; consequently, stable conventional OR estimates could not be reliably obtained using standard maximum-likelihood logistic regression. Quasi-complete separation occurs when a predictor is nearly perfectly associated with the outcome, resulting in unstable parameter estimates and preventing reliable estimation of finite ORs.

Variable	OR	95% CI	p-value
Age, per year increase	0.96	0.93-1.00	0.028
Male sex	1.95	0.88-4.32	0.099
Hypoechogenicity	13.74	6.03-31.27	<0.001
Solid or almost entirely solid composition	2.55	1.07-6.10	0.035
Taller-than-wide orientation	34.43	2.55-465.85	0.008
Irregular or lobulated margins	Not reliably estimable	-	-
Microcalcifications	Not reliably estimable	-	-

Diagnostic performance of guideline-based FNAC recommendations

**Figure 3:**
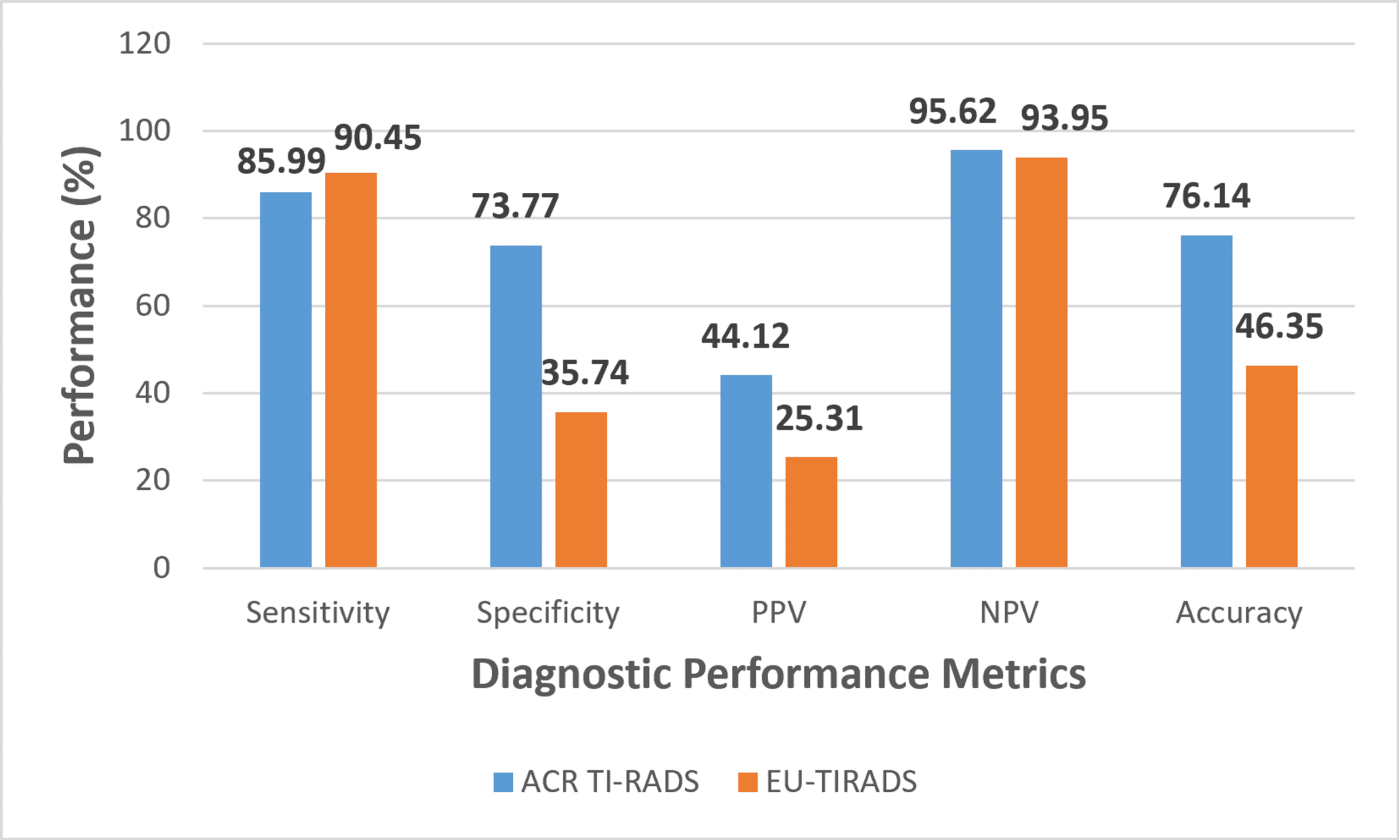
Diagnostic performance of ACR TI-RADS and EU-TI-RADS in the definitive cohort. Bar chart comparing sensitivity, specificity, PPV, NPV, and accuracy. Test positivity was defined as a recommendation for FNAC according to each guideline. Values are shown as point estimates; corresponding 95% CIs are presented in Table 6. ACR TI-RADS, American College of Radiology Thyroid Imaging Reporting and Data System; EU-TI-RADS, European Thyroid Imaging Reporting and Data System; FNAC, fine-needle aspiration cytology; NPV, negative predictive value; PPV, positive predictive value

Using guideline-based FNAC recommendations as the test-positive criterion, ACR TI-RADS demonstrated lower sensitivity but substantially higher specificity than EU-TI-RADS. ACR TI-RADS also demonstrated higher overall accuracy, higher PPV, and a higher DOR. EU-TI-RADS showed marginally higher sensitivity but at the cost of substantially more false-positive FNAC recommendations. These findings are summarized in Table 6 and graphically in Figure 3.

**Table 6:** Diagnostic performance of ACR TI-RADS and EU-TI-RADS for malignancy prediction (n = 809). Values are presented as percentages with 95% CIs in parentheses unless otherwise stated. Diagnostic accuracy represents the proportion of correctly classified nodules. ACR TI-RADS, American College of Radiology Thyroid Imaging Reporting and Data System; DOR, diagnostic OR; EU-TI-RADS, European Thyroid Imaging Reporting and Data System; FNAC, fine-needle aspiration cytology; LR+, positive likelihood ratio; LR-, negative likelihood ratio; NPV, negative predictive value; PPV, positive predictive value

Metric	ACR TI-RADS	EU-TI-RADS
True positive	135	142
False negative	22	15
True negative	481	233
False positive	171	419
Sensitivity	85.99 (95% CI: 79.69-90.56)	90.45 (95% CI: 84.84-94.12)
Specificity	73.77 (95% CI: 70.26-77.00)	35.74 (95% CI: 32.15-39.49)
PPV	44.12 (95% CI: 38.66-49.72)	25.31 (95% CI: 21.89-29.07)
NPV	95.63 (95% CI: 93.47-97.09)	93.95 (95% CI: 90.26-96.30)
Accuracy	76.14 (95% CI: 73.09-78.95)	46.35 (95% CI: 42.94-49.80)
LR+	3.28	1.41
LR-	0.19	0.27
DOR	17.26	5.26

On paired comparison using continuity-corrected McNemar testing, EU-TI-RADS demonstrated slightly higher sensitivity among malignant nodules, identifying eight malignant nodules that were not selected for FNAC by ACR TI-RADS, whereas ACR TI-RADS identified one malignant nodule that was not selected by EU-TI-RADS (McNemar χ² = 4.00, p = 0.046). In contrast, ACR TI-RADS demonstrated substantially higher specificity among benign nodules, with EU-TI-RADS recommending FNAC in 252 benign nodules that were not selected by ACR TI-RADS, compared with only four benign nodules recommended by ACR TI-RADS but not by EU-TI-RADS (McNemar χ² = 238.32, p < 0.001). Overall FNAC recommendation patterns differed significantly between the two systems (McNemar χ² = 243.46, p < 0.001). Ordinal discriminatory performance was excellent for both classification systems, with AUC values of 0.939 for ACR TI-RADS and 0.930 for EU-TI-RADS (Table 7).

**Table 7:** Paired comparison and ordinal discriminatory performance. McNemar testing was used for paired recommendation comparisons because both ACR TI-RADS and EU-TI-RADS were applied to the same nodules. McNemar testing was performed using the continuity-corrected chi-square test. Sensitivity comparison was performed among malignant nodules, specificity comparison was performed among benign nodules, and the overall recommendation comparison was performed across the full definitive cohort. Ordinal AUC values are descriptive and were not assessed using McNemar testing. McNemar χ² statistics were derived from discordant paired counts (shown in the table), and corresponding p-values reflect two-tailed continuity-corrected tests. ACR, American College of Radiology; ACR TI-RADS, American College of Radiology Thyroid Imaging Reporting and Data System; AUC, area under the receiver operating characteristic curve; EU, European; EU-TI-RADS, European Thyroid Imaging Reporting and Data System

Comparison	Discordant pattern/result	Test statistic	p-value
Sensitivity comparison in malignant nodules	EU positive/ACR negative = 8; ACR positive/EU negative = 1	McNemar χ² = 4.00	0.046
Specificity comparison in benign nodules	EU positive/ACR negative = 252; ACR positive/EU negative = 4	McNemar χ² = 238.32	<0.001
Overall recommendation comparison	EU positive/ACR negative = 260; ACR positive/EU negative = 5	McNemar χ² = 243.46	<0.001
Ordinal AUC	ACR 0.939; EU 0.930	Not applicable	Descriptive

Cross-classification between ACR TI-RADS and EU-TI-RADS

Cross-classification demonstrated that most ACR TR2 and TR3 nodules corresponded to EU-TI-RADS 3, whereas most ACR TR5 nodules corresponded to EU-TI-RADS 5. A substantial proportion of ACR TR3 nodules were categorized as EU-TI-RADS 3 and may be recommended differently because of system-specific size thresholds and FNAC criteria (Table 8).

**Table 8:** Cross-classification of ACR TI-RADS and EU-TI-RADS categories in the definitive cohort (n = 809). Values are presented as the number of nodules. ACR TI-RADS, American College of Radiology Thyroid Imaging Reporting and Data System; EU, European; EU-TI-RADS, European Thyroid Imaging Reporting and Data System

ACR TI-RADS	EU 2	EU 3	EU 4	EU 5
TR1	10	0	0	0
TR2	0	317	0	0
TR3	0	287	2	0
TR4	0	34	38	13
TR5	0	0	1	107

Sensitivity analysis for excluded nonoperated Bethesda III/IV nodules

Sensitivity analysis was performed for the 74 excluded nonoperated Bethesda III/IV nodules. When these indeterminate nodules were conservatively classified as benign, ACR TI-RADS retained higher specificity and higher overall accuracy than EU-TI-RADS. When these nodules were classified as malignant, EU-TI-RADS retained higher sensitivity, but ACR TI-RADS remained more specific and showed higher overall accuracy. These analyses support the robustness of the main conclusion that ACR TI-RADS provides greater specificity and potential biopsy reduction, whereas EU-TI-RADS provides greater sensitivity (Table 9).

**Table 9:** Sensitivity analysis including excluded nonoperated Bethesda III/IV nodules. Bethesda I nodules were not included in the sensitivity analysis because they were nondiagnostic. ACR, American College of Radiology; DOR, diagnostic OR; EU, European; NPV, negative predictive value; PPV, positive predictive value

Scenario	System	Sensitivity	Specificity	PPV	NPV	Accuracy	DOR
Bethesda III/IV as benign	ACR	85.99%	71.63%	39.59%	95.94%	74.18%	15.49
Bethesda III/IV as benign	EU	90.45%	35.95%	23.39%	94.57%	45.64%	5.31
Bethesda III/IV as malignant	ACR	73.59%	73.77%	49.85%	88.75%	73.73%	7.84
Bethesda III/IV as malignant	EU	81.39%	35.74%	30.97%	84.42%	47.68%	2.43

Analysis of missed malignancies

Missed malignancies are detailed in Appendix A, which summarizes the sonographic characteristics of malignant nodules not selected for FNAC by ACR TI-RADS, EU-TI-RADS, or both systems. Among 157 malignant nodules, ACR TI-RADS did not recommend FNAC in 22 cases (14.0%), whereas EU-TI-RADS did not recommend FNAC in 15 cases (9.6%). Of these, 14 nodules were missed by both systems, eight were missed by ACR only (but would have been selected by EU-TI-RADS), and one was missed by EU-TI-RADS only (but would have been selected by ACR TI-RADS). McNemar testing demonstrated a modest but statistically significant difference in sensitivity between systems (χ² = 4.00, p = 0.046). These findings indicate that although EU-TI-RADS missed fewer cancers (15 vs 22), the absolute difference was small; however, in paired comparison, EU-TI-RADS showed higher sensitivity in this cohort. Malignancies not selected for FNAC by one system were reviewed for imaging characteristics, including nodule size, echogenicity, composition, margin features, and calcification pattern.

## Discussion

This study provides a pragmatic, decision-level comparison of ACR TI-RADS and EU-TI-RADS using guideline-based FNAC recommendation as the diagnostic threshold in a real-world primary healthcare cohort. In the definitive cohort of 809 nodules, the malignancy prevalence of 19.4% falls within the range reported in previous mixed surgical and cytology-based series, supporting the external validity of the dataset [11]. The predominance of benign nodules reflects the known epidemiology of thyroid disease [12], while the higher proportion of malignant nodules observed among male patients is in keeping with prior reports demonstrating increased risk and more aggressive disease characteristics in this subgroup [13].

Both systems demonstrated high sensitivity and high NPV, supporting their usefulness in excluding malignancy when biopsy is not recommended. However, the systems differed in their diagnostic trade-off: EU-TI-RADS achieved slightly higher sensitivity, whereas ACR TI-RADS demonstrated substantially higher specificity, diagnostic accuracy, PPV, and DOR.

The main practical implication of these findings is that the two systems emphasize different trade-offs. EU-TI-RADS showed marginally higher sensitivity, which may be advantageous when the primary objective is to minimize missed malignancy. However, this was associated with much lower specificity and a larger number of false-positive FNAC recommendations. In contrast, ACR TI-RADS achieved substantially higher specificity and overall accuracy, suggesting that it may be more efficient in routine primary healthcare practice where avoidance of unnecessary biopsy is clinically and operationally important. The higher sensitivity observed with EU-TI-RADS may be advantageous in high-risk patient populations where minimizing missed malignancies is a priority. Conversely, the higher specificity of ACR TI-RADS may reduce unnecessary biopsies and associated healthcare costs.

The paired comparison analysis demonstrated that the principal difference between ACR TI-RADS and EU-TI-RADS lies in biopsy recommendation thresholds rather than overall discriminatory capability. Although EU-TI-RADS achieved a statistically significant increase in sensitivity, the absolute benefit was modest, corresponding to eight additional malignant nodules identified by EU-TI-RADS compared with one additional malignancy identified by ACR TI-RADS. In contrast, the difference in specificity was substantially larger, with ACR TI-RADS avoiding FNAC recommendation in 252 benign nodules that would have undergone biopsy under EU-TI-RADS. These findings indicate that EU-TI-RADS achieves slightly greater cancer detection at the cost of a markedly increased number of unnecessary biopsies.

Importantly, the nearly identical AUC values observed for ACR TI-RADS (0.939) and EU-TI-RADS (0.930) indicate that both systems have an excellent ability to discriminate between benign and malignant nodules. Therefore, the observed differences in clinical performance are not primarily attributable to superior diagnostic discrimination by either system, but rather to different thresholds for recommending FNAC. This finding supports the concept that ACR TI-RADS emphasizes biopsy efficiency and reduction of unnecessary FNAC procedures, whereas EU-TI-RADS prioritizes sensitivity and cancer detection.

Our findings are generally consistent with previous comparative studies demonstrating greater sensitivity for EU-TI-RADS and greater specificity for ACR TI-RADS, although the magnitude of these differences varies across populations and study designs [7,8,14-17]. Table 10 summarizes sensitivity and specificity values across studies, reinforcing the reproducibility of these trends.

**Table 10:** Comparison of the diagnostic performance of ACR TI-RADS and EU-TI-RADS with previously published studies. Reported values are derived from the respective published studies. Direct comparison between studies should be interpreted with caution due to heterogeneity in study design, reference standards, and FNAC thresholds. ACR TI-RADS, American College of Radiology Thyroid Imaging Reporting and Data System; EU-TI-RADS, European Thyroid Imaging Reporting and Data System; FNAC, fine-needle aspiration cytology

Study	ACR TI-RADS sensitivity (%)	ACR TI-RADS specificity (%)	EU-TI-RADS sensitivity (%)	EU-TI-RADS specificity (%)
Yoon et al. [7]	77.3	67.7	87.4	38.9
Grani et al. [8]	83.3	56.2	86.1	32
Na et al. [14]	79.6	65.2	88.3	33.4
Tobcu et al. [15]	78.8	58.4	90.4	48.6
Mohan et al. [16]	93.3	50.8	95.5	26.7
Huh et al. [17]	80.4	62.2	95.2	28.1
Current study	85.99	73.77	90.45	35.74

Evaluation across TI-RADS categories further supports the validity of both stratification approaches. High-risk categories were associated with higher FNAC recommendation rates and malignancy yield, while malignancy was uncommon in low-risk categories. Mid-tier categories accounted for the greatest variability in FNAC utilization and therefore represent the principal area where guideline differences influence clinical workload.

A proportion of nodules classified as low risk nevertheless proceeded to surgery, indicating that management decisions were influenced by factors beyond ultrasound morphology alone.

**Figure 4:**
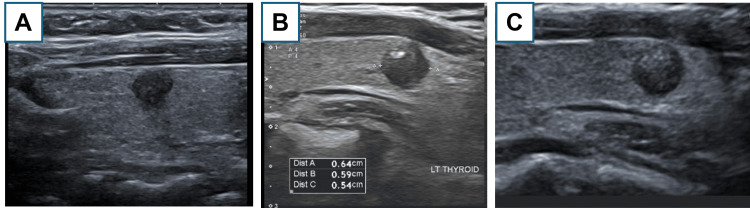
Representative ultrasound images (A-C) of subcentimeter (<1 cm) malignant thyroid nodules that were not recommended for FNAC by either ACR TI-RADS or EU-TI-RADS because they did not meet size-based biopsy thresholds. Despite their small size, these nodules demonstrate suspicious sonographic features, including hypoechogenicity, punctate echogenic foci, and/or irregular margins, illustrating the potential for size-based under-triage. ACR TI-RADS, American College of Radiology Thyroid Imaging Reporting and Data System; EU-TI-RADS, European Thyroid Imaging Reporting and Data System; FNAC, fine-needle aspiration cytology

**Figure 5:**
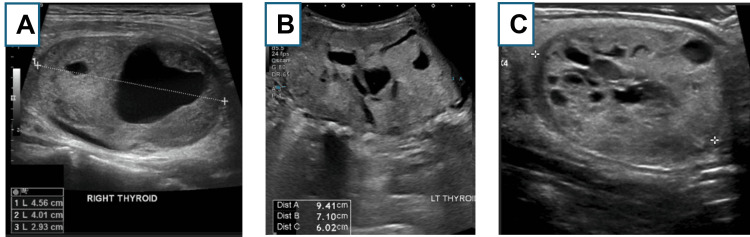
Ultrasound images (A-C), all obtained in sagittal orientation, of large (≥2.5 cm) malignant thyroid nodules with benign-appearing morphology that were not selected for FNAC by ACR TI-RADS but were appropriately identified by EU-TI-RADS. These nodules demonstrate isoechogenicity, oval shape, wider-than-tall orientation, smooth or ill-defined margins, and mixed solid-cystic composition, resulting in lower point-based risk assignment despite malignant histopathology. ACR TI-RADS, American College of Radiology Thyroid Imaging Reporting and Data System; EU-TI-RADS, European Thyroid Imaging Reporting and Data System; FNAC, fine-needle aspiration cytology

Analysis of false-negative malignancies demonstrates that missed cancers predominantly reflect threshold effects rather than systematic misclassification of sonographic features. Among the 14 nodules missed by both systems, 13 were small nodules, commonly <1.5 cm. All 13 small nodules underwent surgical excision and were confirmed as malignant on histopathology (papillary thyroid carcinoma); nine were also classified as Bethesda V/VI on FNAC. Figure 4 demonstrates subcentimeter malignant nodules with high-risk sonographic features that were not recommended for biopsy by either system because minimum size criteria were not met. In contrast, Figure 5 illustrates large malignant nodules with predominantly benign‑appearing morphology that were not selected for FNAC by ACR TI-RADS owing to low point-based risk assignment but were identified by EU-TI-RADS. Collectively, these findings suggest that most missed malignancies resulted from the application of guideline size thresholds rather than failure to identify suspicious sonographic morphology. In this cohort, most missed nodules were either small or lacked overtly aggressive imaging features, which may mitigate clinical impact.

EU-TI-RADS demonstrated a lower false‑negative rate overall, whereas ACR TI-RADS did not recommend FNAC for a small number of malignant nodules measuring ≥2.5 cm. This pattern likely reflects the greater reliance of ACR TI-RADS on weighted pattern‑based scoring. Although infrequent, such cases indicate that clinically relevant malignancies may occasionally fall below selection thresholds when strict size cutoffs are applied. Selective FNAC or short‑interval ultrasound follow‑up for nodules exhibiting at least one high-risk feature may therefore be considered in appropriate clinical contexts, as discussed in prior guideline comparisons.

A clearer distinction between systems emerged in the subgroup of large (≥2.5 cm) malignant nodules not recommended for FNAC by ACR TI-RADS but selected by EU-TI-RADS. These cases were uncommon and are compatible with recognized limitations of pattern-based systems in some follicular‑pattern malignancies. All six nodules demonstrated benign-appearing sonographic features, including isoechogenicity, oval shape, wider-than-tall orientation, mixed solid-cystic composition, and absence of microcalcifications, and had nonsuspicious cytology (Bethesda II (five cases) and III (one case)). Histopathology confirmed a spectrum of follicular pattern tumors, which are recognized to lack classic high-suspicion ultrasound features and therefore accrue fewer points in pattern-based systems such as ACR TI-RADS. By applying broader high-risk criteria and lower FNAC thresholds for indeterminate patterns, EU-TI-RADS identified all six lesions, consistent with prior comparative studies reporting increased sensitivity at the cost of higher biopsy rates [7,8,14-17].

From a service delivery perspective, ACR TI-RADS achieved a higher malignancy yield among biopsied nodules and a substantially lower negative FNAC rate, indicating more efficient utilization of biopsy resources in this cohort. In contrast, EU-TI-RADS reduced the absolute number of missed malignancies but did so through a markedly expanded FNAC volume with lower yield efficiency. Similar trade-offs have been reported in previous comparative studies [8,14], and the balance between sensitivity and workload is likely to depend on institutional priorities and baseline disease prevalence.

Overall, these findings indicate that both ACR TI-RADS and EU-TI-RADS provide clinically useful risk stratification when FNAC recommendation is used as the diagnostic threshold, but they differ in the balance between sensitivity and biopsy efficiency. The observed patterns of false-negative malignancy support careful consideration of selective deviation from rigid size-based thresholds in individualized scenarios, particularly when imaging features raise concern, but guideline size criteria are not met.

Regarding the sonographic features of benign and malignant nodules, our findings confirm that malignant thyroid nodules were more frequently solid, hypoechoic (particularly markedly hypoechoic), associated with irregular or lobulated margins, taller-than-wide orientation, microcalcifications, and extrathyroidal extension with statistically significant differences (Table 4). The multivariable logistic regression analysis further supports the relevance of classic suspicious ultrasound features in malignancy prediction. Hypoechogenicity, solid or almost entirely solid composition, and taller-than-wide orientation remained independently associated with malignancy after adjustment for other demographic and sonographic variables. These findings support the biological and imaging rationale underlying both ACR TI-RADS and EU-TI-RADS, as each system incorporates hypoechogenicity, predominantly solid composition, and taller-than-wide orientation as key indicators of malignancy risk.

The inability to generate stable conventional OR estimates for irregular or lobulated margins and microcalcifications should not be interpreted as evidence of limited clinical significance. Rather, these features were so strongly concentrated among malignant nodules that conventional logistic regression could not reliably estimate their effect size.

Overall, the regression analysis complements the primary diagnostic accuracy comparison by showing that the observed malignancy patterns were not driven only by TI-RADS category assignment but were also supported by independent associations between key sonographic features and final malignant outcome. Among the estimable variables, taller-than-wide orientation demonstrated the strongest independent association with malignancy, followed by hypoechogenicity. These findings further support the sonographic weighting used in both ACR TI-RADS and EU-TI-RADS.

These findings correlated well with previous studies, which suggest that not a single sonographic feature, but a combination of features, more accurately predicts the risk of malignancy [3,18]. These characteristics are consistent with risk stratification criteria outlined in both ACR TI-RADS and EU-TI-RADS. Both systems recognize solid and hypoechoic nodules as high-risk features. EU-TI-RADS places greater emphasis on marked hypoechogenicity, while ACR TI-RADS assigns incremental points for solid composition and echogenicity, allowing finer risk gradation. Taller-than-wide orientation and irregular or lobulated margins are considered major suspicious features in both guidelines. ACR TI-RADS uses a point-based approach, whereas EU-TI-RADS applies categorical thresholds, which may simplify clinical decision-making but reduce granularity. Microcalcifications are a critical predictor in both systems. ACR TI-RADS differentiates between punctate echogenic foci and macrocalcifications, while EU-TI-RADS primarily highlights microcalcifications as a high-risk marker. Absence of a halo and presence of extrathyroidal extension, though less frequent, remain important indicators of malignancy and are acknowledged in both frameworks.

Strengths

This study has several strengths. First, all nodule images and imaging criteria were retrospectively reviewed by three consultant radiologists with 15-20 years of thyroid imaging experience. Second, reviewers were blinded to cytological and histopathological outcomes, which reduces interpretation bias. Third, disagreements were resolved by consensus, improving consistency of final categorization. Fourth, the study reflects a real-world primary healthcare population rather than a highly selected tertiary referral cohort. Finally, the study used a predefined composite reference standard and direct paired comparison of two classification systems applied to the same nodules. Moreover, the diagnostic pathway reflects direct referral from primary care health centers to a secondary hospital for FNAC, representing a realistic primary-to-secondary care flow that enhances the generalizability of the findings beyond tertiary center cohorts.

Limitations

Several limitations should be acknowledged. The retrospective study design may introduce selection bias. The study was conducted within one healthcare system, which may limit generalizability to other populations and practice settings. A limitation of this study is the exclusion of nonoperated nodules with Bethesda III and IV cytology and Bethesda I nondiagnostic aspirates from the primary diagnostic accuracy analysis because definitive benign or malignant classification could not be established. Excluding nonoperated Bethesda III and IV nodules may have enriched the primary analysis cohort for more definitive benign and malignant outcomes and may not fully reflect the diagnostic uncertainty encountered in routine cytology practice. However, sensitivity analyses incorporating these nodules under alternative assumptions produced similar comparative results, supporting the robustness of the primary findings. Although three experienced radiologists reviewed the images independently and reached consensus in cases of disagreement, formal interobserver agreement statistics (e.g., Cohen’s kappa or Fleiss’ kappa) were not available because individual reader-level classifications were not retained in the study dataset. Future prospective studies should evaluate the interobserver reproducibility of both classification systems. Because the unit of analysis was the nodule rather than the patient, individuals with more than one eligible biopsied nodule could contribute more than one observation. Within-patient clustering was not explicitly modelled and should be considered when interpreting inferential estimates. In addition, nonoperated benign nodules classified by cytology may still carry a small residual risk of false-negative cytology, particularly in the absence of long-term follow-up. Despite these limitations, the study reflects real-world clinical practice, uses clearly defined diagnostic thresholds, and provides a pragmatic comparison of two widely adopted TI-RADS systems using guideline-based FNAC recommendations.

## Conclusions

In this primary healthcare cohort, both ACR TI-RADS and EU-TI-RADS demonstrated high sensitivity and high NPV for thyroid malignancy. EU-TI-RADS provided slightly higher sensitivity, whereas ACR TI-RADS demonstrated substantially higher specificity, overall accuracy, and DOR. These findings suggest that ACR TI-RADS may be preferable when reducing unnecessary FNAC is a priority, whereas EU-TI-RADS may be favored when maximizing sensitivity is the primary objective. Multivariable analysis demonstrated that hypoechogenicity, solid or almost entirely solid composition, and taller-than-wide orientation were independent predictors of malignancy. Irregular or lobulated margins and microcalcifications were also strongly associated with malignancy, although stable conventional OR estimates could not be reliably derived because of quasi-complete separation. Despite similar overall discriminatory performance, ACR TI-RADS substantially reduced unnecessary FNAC recommendations, whereas EU-TI-RADS achieved a modest increase in sensitivity at the cost of considerably higher biopsy utilization.

## Appendices

Appendix A 

**Table 11:** Sonographic characteristics of malignant nodules not selected for FNAC by ACR TI-RADS, EU-TI-RADS, or both systems. Size categories are as recorded in the dataset (e.g., 0.5-<1 cm, 1.0-<1.5 cm, etc.). “Missed by system” indicates malignant nodules for which the specified guideline did not recommend FNAC. Most missed malignancies were subcentimeter TR5 nodules or large mixed nodules with low point-based risk. ACR, American College of Radiology; ACR TI-RADS, American College of Radiology Thyroid Imaging Reporting and Data System; EU, European; EU-TI-RADS, European Thyroid Imaging Reporting and Data System; FNAC, fine-needle aspiration cytology

Case number	Size (cm category)	Composition	Echogenicity	Calcification	Orientation	Margin	ACR TI-RADS	EU-TI-RADS	Missed by system
1	1.5-2.0 cm	Mixed solid-cystic	Hypoechoic	Absent	Wider-than-tall	Smooth	TR3	EU-4	ACR only
2	0.5-<1 cm	Solid	Markedly hypoechoic	Micro-calcification	Taller-than-wide	Irregular	TR5	EU-5	Both
3	1.0-<1.5 cm	Solid	Isoechoic	Absent	Wider-than-tall	Smooth	TR3	EU-3	Both
4	0.5-<1 cm	Solid	Markedly hypoechoic	Absent	Wider-than-tall	Irregular	TR5	EU-5	Both
5	1.0-<1.5 cm	Solid	Isoechoic	Rim-calcification	Wider-than-tall	Smooth	TR4	EU-3	Both
6	0.5-<1 cm	Solid	Markedly hypoechoic	Micro and macro-calcification	Wider-than-tall	Irregular	TR5	EU-5	Both
7	1.5-2.0 cm	Solid	Isoechoic	Macro-calcification	Wider-than-tall	Ill-defined	TR4	EU-3	EU only
8	0.5-<1 cm	Solid	Hypoechoic	Absent	Wider-than-tall	Ill-defined	TR4	EU-4	Both
9	0.5-<1 cm	Solid	Markedly hypoechoic	Micro-calcification	Wider-than-tall	Smooth	TR5	EU-5	Both
10	>=2.5 cm	Mixed solid-cystic	Isoechoic	Absent	Wider-than-tall	Smooth	TR2	EU-3	ACR only
11	0.5-<1 cm	Solid	Hypoechoic	Absent	Wider-than-tall	Smooth	TR4	EU-4	Both
12	1.0-<1.5 cm	Mixed solid-cystic	Hypoechoic	Micro-calcification	Wider-than-tall	Ill-defined	TR4	EU-5	ACR only
13	0.5-<1 cm	Solid	Hypoechoic	Micro-calcification	Taller-than-wide	Irregular	TR5	EU-5	Both
14	>=2.5 cm	Mixed solid-cystic	Isoechoic	Absent	Wider-than-tall	Ill-defined	TR2	EU-3	ACR only
15	>=2.5 cm	Mixed solid-cystic	Isoechoic	Absent	Wider-than-tall	Ill-defined	TR2	EU-3	ACR only
16	0.5-<1 cm	Solid	Hypoechoic	Absent	Wider-than-tall	Smooth	TR4	EU-4	Both
17	0.5-<1 cm	Solid	Hypoechoic	Micro and rim-calcification	Wider-than-tall	Irregular	TR5	EU-5	Both
18	0.5-<1 cm	Solid	Hypoechoic	Micro and macro-calcification	Wider-than-tall	Smooth	TR5	EU-5	Both
19	0.5-<1 cm	Solid	Hypoechoic	Micro-calcification	Wider-than-tall	Irregular	TR5	EU-5	Both
20	>=2.5 cm	Mixed solid-cystic	Isoechoic	Absent	Wider-than-tall	Smooth	TR2	EU-3	ACR only
21	0.5-<1 cm	Solid	Hypoechoic	Macro and rim-calcification	Taller-than-wide	Ill-defined	TR5	EU-5	Both
22	>=2.5 cm	Mixed solid-cystic	Isoechoic	Absent	Wider-than-tall	Smooth	TR2	EU-3	ACR only
23	>=2.5 cm	Mixed solid-cystic	Isoechoic	Absent	Wider-than-tall	Smooth	TR2	EU-3	ACR only
